# Overview of the Neuroprotective Effects of the MAO-Inhibiting Antidepressant Phenelzine

**DOI:** 10.1007/s10571-021-01078-3

**Published:** 2021-04-10

**Authors:** Dmitriy Matveychuk, Erin M. MacKenzie, David Kumpula, Mee-Sook Song, Andrew Holt, Satyabrata Kar, Kathryn G. Todd, Paul L. Wood, Glen B. Baker

**Affiliations:** 1 Department of Psychiatry (Neurochemical Research Unit), University of Alberta, 12-105B Clinical Sciences Building, Edmonton, AB T6G 2G3 Canada; 2 Present Address: Altos Biologics, Seoul, South Korea; 3 Department of Medicine (Neurology), University of Alberta, Edmonton, Canada; 4 College of Veterinary Medicine, Lincoln Memorial University, Harrogate, TN USA

**Keywords:** Phenelzine, Monoamine oxidase, β-Phenylethylidenehydrazine, Neuroprotection, γ-Aminobutyric acid, Reactive aldehydes

## Abstract

Phenelzine (PLZ) is a monoamine oxidase (MAO)-inhibiting antidepressant with anxiolytic properties. This multifaceted drug has a number of pharmacological and neurochemical effects in addition to inhibition of MAO, and findings on these effects have contributed to a body of evidence indicating that PLZ also has neuroprotective/neurorescue properties. These attributes are reviewed in this paper and include catabolism to the active metabolite β-phenylethylidenehydrazine (PEH) and effects of PLZ and PEH on the GABA-glutamate balance in brain, sequestration of reactive aldehydes, and inhibition of primary amine oxidase. Also discussed are the encouraging findings of the effects of PLZ in animal models of stroke, spinal cord injury, traumatic brain injury, and multiple sclerosis, as well other actions such as reduction of nitrative stress, reduction of the effects of a toxin on dopaminergic neurons, potential anticonvulsant actions, and effects on brain-derived neurotrophic factor, neural cell adhesion molecules, an anti-apoptotic factor, and brain levels of ornithine and *N*-acetylamino acids.

## Introduction

In the last 25 years, numerous exciting research reports have demonstrated that many antidepressants and antipsychotics have neuroprotective and/or neurorescue properties (Baker et al. 2012; Chen and Nasrallah 2019; Hunsberger et al. 2009; Li and Xu 2007; Lieberman et al. 2008; Shadfar et al. 2018; Song et al. 2013; Sowa et al. 2004; Tatton et al. 2003; Xu et al. 2003; Youdim and Bakhle 2006; Young 2002). With regard to monoamine oxidase inhibitors (MAOIs), much of this research has focused on the selective irreversible MAO-B inhibitors l-deprenyl and rasagaline, which have demonstrated neuroprotective properties in a wide variety of models in vitro and in vivo (Gerlach et al. 1996; Hill et al. 2020; Magyar and Szende 2004; Sowa et al. 2004; Szökő et al. 2018; Tatton et al. 2003; Youdim et al. 2006). However, there is also extensive research demonstrating multiple actions of the MAOI phenelzine (PLZ) that may contribute to neuroprotection/neurorescue, and the focus of the current review paper is on PLZ and its numerous properties in that regard.

**Fig. 1:**
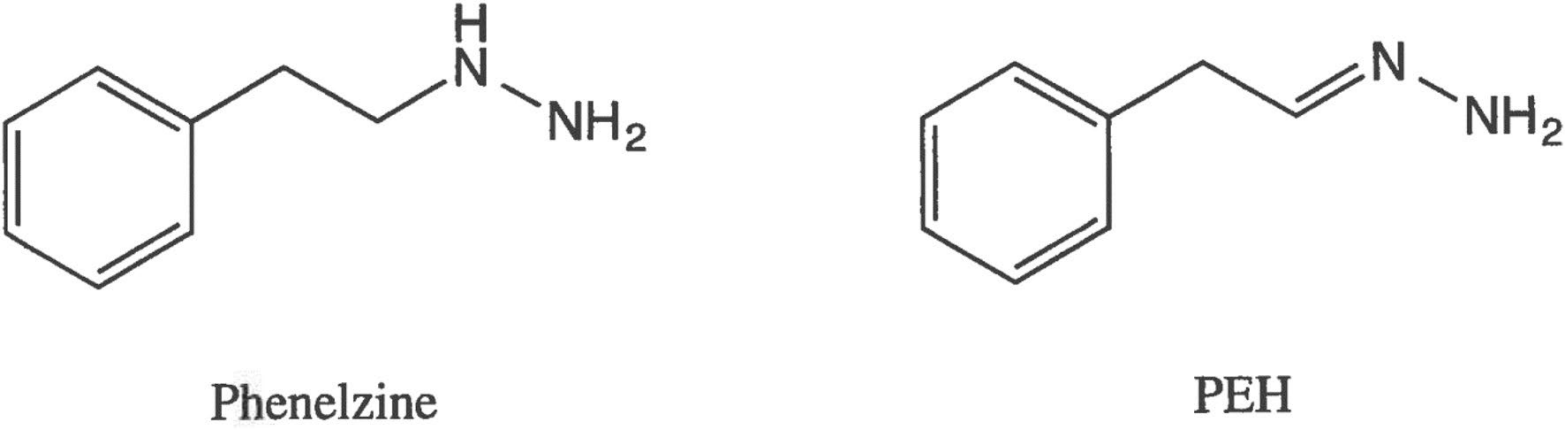
Chemical structures of PLZ and PEH

PLZ (Fig. 1) is a non-selective (inhibits both MAO-A and -B) irreversible MAOI marketed as an antidepressant, but it has also been reported in clinical studies to be effective in treatment of anxiety disorders such as panic disorder and social anxiety disorder (Aarre 2003; Buigues and Vallejo 1987; Davidson et al. 1987; Liebowitz et al. 1988; McGrath et al. 1986; Sheehan et al. 1980; Williams et al. 2020; Zhang and Davidson 2007). As with other irreversible non-subtype-selective MAO inhibitors, some dietary restrictions are recommended for patients on PLZ to avoid a potential hypertensive crisis when certain foods are ingested, although effects on blood pressure appear to be less problematic than originally proposed in the literature (see Gillman 2018 for discussion of this aspect). PLZ is a multifaceted drug that acts on several enzymes and other factors proposed to be involved in neuroprotection and in the etiology of a variety of psychiatric and neurological disorders (Al-Nuaimi et al. 2012; Baker et al. 1991, 2019; Hill et al. 2020; Holt et al. 2004; Jarrahi et al. 2020; MacKenzie et al. 2010; Popov and Matthies 1969; Ribaudo et al. 2020; Song et al. 2010; Wood et al. 2006b). The following aspects which appear to be contributing to the neuroprotective effects of PLZ will be discussed in detail in this review: contribution of an active metabolite; inhibition of MAO; inhibition of γ-aminobutyric acid transaminase (GABA-T) and elevation of brain GABA levels; elevation of brain levels of the amino acid ornithine and *N*-acetylamino acids; sequestration of toxic reactive aldehydes such as formaldehyde, acrolein, 3-aminopropanal, malondialdehyde, and 4-hydroxy-2-nonenal (4-HNE); and inhibition of primary amine oxidase [PrAO, also known as semicarbazide-sensitive amine oxidase (SSAO)]. Some recent findings on additional factors that may be involved in its neuroprotective actions will also be discussed.

In preparation for this review paper, the Web of Science databases and PubMed were reviewed for papers from the years 1995 to 2020. The following search headings were used: phenelzine and neuroprotection; phenelzine and oxidative stress; reactive aldehydes in psychiatric disorders; reactive aldehydes in neurological disorders; acrolein in disease states; semicarbazide-sensitive amine oxidase in disease states; semicarbazide-sensitive amine oxidase in Alzheimer’s disease; and primary amine oxidase in Alzheimer’s disease. The reference lists in a number of papers on the above topics were also searched for additional appropriate references, and we have also included results from our own investigations on PLZ.

## β-Phenylethylidenehydrazine (PEH), an Active Metabolite of PLZ

PLZ is metabolized extensively, with metabolites including β-phenylethylamine, phenylacetic acid, *p*-hydroxyphenylacetic acid, β-phenylethylidenehydrazine (PEH), and phenylethyldiazene (Clineschmidt and Horita 1969a, 1969b; Kennedy et al. 2009; Kumpula et al. 2010; Robinson et al. 1985; Tipton 1972; Tipton and Spires 1972). Of these, PEH (Fig. 1) appears to contribute markedly to the neuroprotective effects of the parent drug. In addition to being inhibited by PLZ, MAO also catalyzes the catabolism of PLZ (Clineschmidt and Horita 1969a, 1969b; Horita 1965; Popov and Matthies 1969; Tipton 1971, 1972), with PEH being a prominent metabolite (Patek and Hellerman 1974; Tipton and Spires 1972). Although PEH is only a weak inhibitor of MAO (MacKenzie et al. 2008a; Matveychuk 2015; Matveychuk et al. 2013; Paslawski et al. 2001), it has several actions, including effects on brain levels of GABA, sequestration of reactive aldehydes, and inhibition of PrAO that may contribute to the neuroprotective/neurorescue properties of PLZ, and these actions will be described in the appropriate sections below.

## Inhibition of MAO-A and MAO-B

By inhibiting MAO, PLZ elevates brain levels of the monoamine neurotransmitters 5-hydroxytryptamine (5-HT, serotonin), noradrenaline, and dopamine which have been proposed to be functionally deficient in depression (reviews: Baker and Dewhurst 1985; Blier 2016). However, the inhibition of MAO may also be associated with some of the neuroprotective properties of PLZ. The catalytic cycle of MAO results in the production of H_2_O_2_, an aldehyde (via an imine) and ammonia (for primary amines) or an alkyl-substituted amine (for secondary and tertiary amines). Ammonia, H_2_O_2_, and some of the aldehyde metabolites formed are potentially neurotoxic (Marchitti et al. 2007; Wang et al. 2004; Wood et al. 2007a; Yang 2004; Yang et al. 2003), and their production is reduced by MAOIs. The MAO-catalyzed oxidation of catecholamines and 5-HT results in formation of 3,4-dihydroxyphenylacetaldehyde (DOPAL) from dopamine, 3,4-dihydroxyphenylglycolaldehyde (DOPEGAL) from noradrenaline and adrenaline, and 5-hydroxyindoleacetaldehyde (5-HIAL) from 5-HT. These three aldehydes have been reported to produce toxicity in a variety of in vitro and in vivo experiments (Cagle et al. 2019; Eisenhofer et al. 2004; Marchitti et al. 2007) and have been implicated in the etiology of Alzheimer’s disease (AD) and Parkinson’s disease (PD) (Burke et al. 2003, 2004; Grünblatt et al. 2004; Masato et al. 2019; Panneton et al. 2010).

It has been reported that with aging in humans there is increased brain activity of MAO-B, but not of MAO-A (Fowler et al. 1997, 2002; Shemyakov 2001; Volchegorskii et al. 2001). The activity of MAO-B is also increased in brains of AD patients relative to age-matched controls, while MAO-A activity has been reported to be unchanged or increased depending on the brain regions under investigation (Adolfsson et al. 1980; Jossan et al. 1991; Oreland and Gottfries 1986; Quartey et al. 2018; Reinikainen et al. 1988; Saura et al. 1994; Sherif et al. 1992; Sparks et al. 1991). The increased MAO-B activity in aging and AD may be the result of age- and neurodegeneration-related proliferation of glial cells since MAO-B is expressed in glia (Beach et al. 1989; Liu et al. 1996; Riederer et al. 1987). This increase in MAO-B activity has been proposed to contribute to destruction of cholinergic neurons, cognitive dysfunction, and formation of amyloid plaques and neurofilbrillary tangles (Cai 2014; Manzoor and Hoda 2020; Schedin-Weiss et al. 2017). The mechanism of the effect of MAO-B on cholinergic neurons is unclear, but may be the result of the excess MAO-B producing increased levels of hydrogen peroxide, subsequently leading to formation of reactive oxygen species such as the hydroxyl radical (Practico 2008; Quartey et al., 2018; Riederer et al. 2004; Sturm et al. 2017; Sa et al. 2019). Interestingly, Jossan et al (1989), in a study with a cholinergic neurotoxin in rats reported that degeneration of cholinergic neurons also results in an increase in activity of MAO-B, but not of MAO-A, in hippocampus, striatum, and cortex. These authors suggested that the increase was due to increased gliosis after cholinergic neuronal degeneration and that increased MAO-B activity may reflect degeneration of the cholinergic system. It has also been proposed that MAO-B may increase neurodegeneration via regulation of β-amyloid levels by activating γ-secretase in neurons (Schedin-Weiss et al. 2017). An interaction of MAO-B with GABA may also contribute to the cognitive dysfunction seen in AD. MAO-B has been reported to catalyze formation of GABA from the polyamine putrescine in glia, and the GABA thus formed is released to mediate tonic inhibition (Yoon et al. 2014). The presence of reactive astrocytes in close proximity to amyloid plaques has been observed in AD, and it has been proposed that aberrant levels of GABA formed by the action of MAO-B in such astrocytes impair memory in mouse models of AD (Jo et al. 2014).

MAOIs would attenuate the effects of increased MAO-B, although they have not been utilized extensively in treatment of AD; such studies have been conducted mainly with irreversible MAO-B inhibitors such as selegiline and rasagiline, and the results in long-term studies have been generally disappointing (Park et al., 2019; Schneider et al. 2014; Tabi et al. 2020). Park et al. (2019) proposed that the MAO-B/GABA interaction may account for why the irreversible MAO-B-selective inhibitor l-deprenyl (selegiline) has been found to improve cognitive deficits in AD after short-term, but not long-term, administration. In studies on the amyloid precursor, protein/presenilin 1 (APP/PS1) mouse model of AD, these researchers found that selegiline reduced the aberrant levels of GABA initially by inhibiting MAO-B but that increased activity of the compensatory GABA-synthesizing enzyme diamine oxidase after longer administration of selegiline resulted in increased levels of GABA again; they found that a highly selective, reversible MAO-B inhibitor (KDS2010) did not have this effect and reversed learning and memory impairment in this mouse model (Park et al 2019). Because of their effects on reactive oxygen species, toxic aldehydes, and PrAO (see the following discussions in this review), PLZ and PEH might be useful adjunctive drugs to study in AD, although their GABA-enhancing effects (see next section) may contribute to memory impairment (Park et al. 2019).

## Elevation of Brain Levels of GABA by PLZ and PEH

**Fig. 2:**
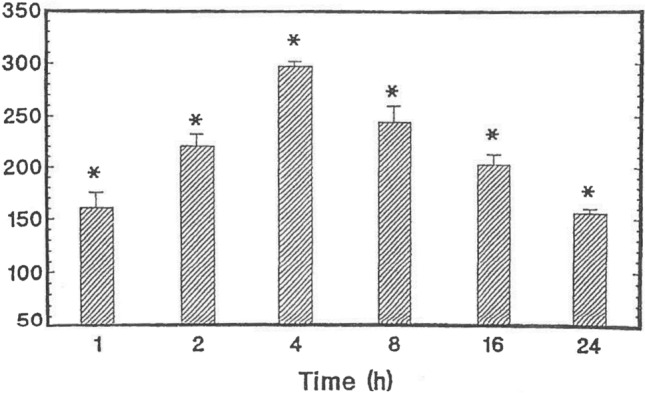
Effects of PLZ (15 mg/kg ip) on rat whole brain levels of GABA (from Baker et al. 1991 with permission from Elsevier). Values represent mean % of controls ± SEM (*N* = 5). Control GABA values = 234 ± 8 μg/g (*N* = 30). *p < 0.05 compared to control values

Although it was developed as an MAOI, it is well documented that PLZ also causes an elevation of brain levels of GABA in rats (Baker et al. 1991; McKenna et al. 1994; Paslawski et al. 1995; Popov and Matthies 1969; Todd and Baker 1995; Fig. 2). In 1969 Popov and Matthies reported that the treatment of rats with another MAOI before administering PLZ attenuated the ability of PLZ to elevate brain levels of GABA, suggesting that a metabolite formed by the action of MAO on PLZ was responsible for the observed effects. Since PEH had been reported as a metabolite of PLZ (Patek and Hellerman 1974; Tipton and Spires 1972), we synthesized PEH, which differs structurally from PLZ in the presence of a double bond. We found that PEH, like PLZ, caused a rapid, marked, and relatively long-lasting elevation of brain GABA after a single intraperitoneal (ip) injection to rats (MacKenzie et al. 2010; Paslawski et al. 2001). While PEH is a weak MAO inhibitor (MacKenzie et al. 2010; Matveychuk et al. 2013; Paslawski et al. 2001), this metabolite inhibits GABA transaminase (GABA-T) (Paslawski et al. 2001; MacKenzie et al. 2008a), presumably contributing to the GABA-elevating action of PLZ.

**Fig. 3:**
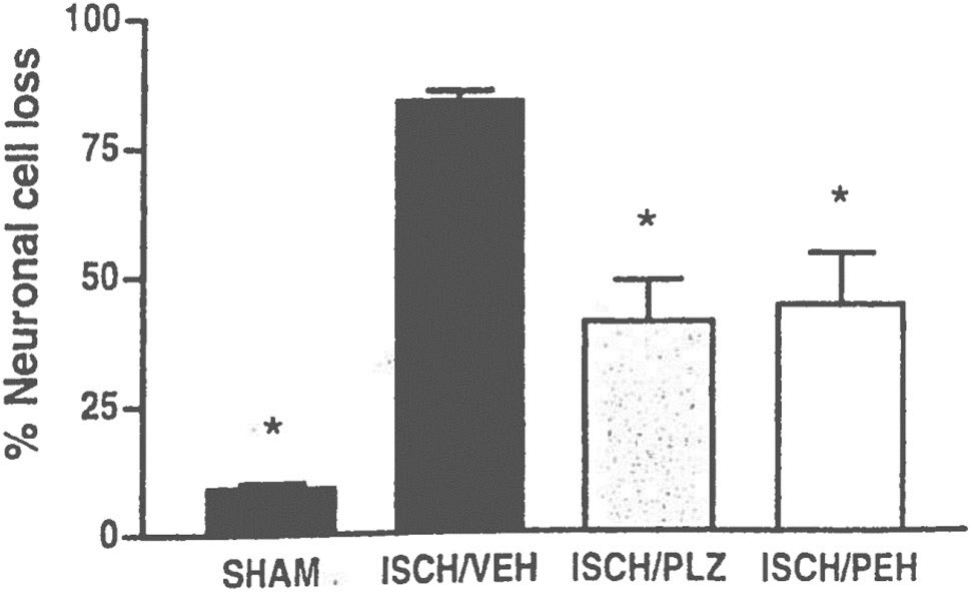
Attenuation by PLZ and PEH of neuronal loss in a global ischemia model of stroke (carotid ligation for 5 min followed by reperfusion). PLZ (15 mg/kg ip) or PEH (30 mg/kg ip) were started 3 h post reperfusion. Gerbils were treated once daily at the same dose for 6 days and were euthanized 24 h after the last dose. Neuronal cell counts were obtained in the hippocampal CA1 region. *p < 0.05 compared to values in the ischemia-vehicle gerbils. *N* = 6 for drug-treated gerbils and *N* = 12 for SHAM and ISCH/VEH gerbils

Our interest in possible neuroprotective/neurorescue actions of PLZ and PEH was stimulated by reports that various GABAergic drugs decreased neuronal cell loss in animal models of stroke (global and focal ischemia) (Chen Xu et al. 2000; Leker and Neufeld 2003; Shuaib et al. 1992, 1997; Shuaib and Kanthan 1997; Sydserff et al. 2000) and by suggestions that such agents were effective by counteracting the deleterious excitotoxic effects of the increased glutamate release that occurs in stroke (Green et al. 2000; Schwartz-Bloom and Sah 2001; Shuaib and Kanthan 1997; Stumm et al. 2001). Indeed, there is now a large body of literature indicating the importance of maintaining the exquisite balance between GABA and glutamate in the brain and suggesting that a disruption of that balance is a feature of several psychiatric and neurological disorders, including depression, mania, epilepsy, amyotrophic lateral sclerosis, schizophrenia, multiple sclerosis, and stroke (Cohen et al. 2015; Foerster et al. 2013; Green et al. 2000; Ketter and Wang 2003; Kim and Yoon 2017; Luscher et al. 2011; Naylor 2010; Potter et al. 2016; Wassef et al. 2003). We tested PLZ and PEH in a global ischemia model in the gerbil (the animal model that is used primarily in such studies) and found marked neurorescue effects with each of these drugs at doses which caused pronounced increases in brain levels of GABA (Sowa et al. 2003, Sowa et al. 2005; Tanay et al. 2002; Todd et al. 1999; Wood et al. 2006b and Fig. 3).

## Sequestration of Reactive Aldehydes by PLZ and PEH

Another important concept which is of considerable interest with regard to possible neuroprotective mechanisms of action of PLZ, PEH, and structurally related drugs is their attenuation of “aldehyde overload”, i.e., excessive levels of toxic reactive aldehydes (Wood 2006; Wood et al. 2006b). Through Michael addition and/or formation of a Schiff base, aldehydes can form adducts with and cross-link proteins, nucleic acids, and aminophospholipids (Esterbauer et al. 1991), leading to a variety of adverse effects. This may result in inhibition of DNA, RNA, and protein synthesis, disruption of protein and cell membrane function, dysfunction of calcium homeostasis, and interference with pathways regulating cell respiration and glycolysis (Dang et al. 2010; Esterbauer et al. 1991; Lovell et al. 2001; Wood et al. 2006b).

There has been a great deal of research in the last two decades focusing on the role of toxic reactive aldehydes in neurodegeneration (Chen et al. 2016a; Matveychuk et al. 2011; Moghe et al. 2015; Ng et al. 2008; Perluigi et al. 2012; Romano et al. 2017; Sultana et al. 2013; Taso et al. 2019; Xiao et al. 2017). Potential sources of these aldehydes include lipid peroxidation as a result of oxidative stress, carbohydrate autoxidation and metabolism, cytochrome P450-mediated oxidation of alcohols, myeloperoxidase-mediated oxidation of amino acids, and catalytic activity of amine oxidases (O'Brien et al. 2005; Wood et al. 2006a). Production of acrolein, malondialdehyde and 4-hydroxy-2-nonenal (4-HNE) may result from lipid peroxidation (Esterbauer et al. 1991; Lee and Park 2013; Ou et al. 2002; Reed 2011; Uchida et al. 1998; Yadav and Ramana 2013). As mentioned above, DOPAL, DOPEGAL and 5-HIAL result from the oxidation of catecholamines and 5-HT by MAO (Eisenhofer et al. 2004; Marchitti et al. 2007). Several possible enzymes may oxidize polyamines, resulting in generation of 3-aminopropanal (3-AP) and acrolein (Agostinelli et al. 2010; Houen et al. 1994; Wood et al. 2006b, 2007a), and formaldehyde and methylglyoxal are generated by oxidation of methylamine and aminoacetone, respectively, by PrAO (Lyles and Chalmers 1992; Lyles et al. 1990). Accumulation of reactive aldehydes and resultant toxicity can occur because of reduced catabolism by enzymes such as glutathione-*S*-transferase, aldehyde dehydrogenase, and aldo–keto reductase (Ivanova et al. 1998; Wood et al. 2007b). Accumulation of reactive aldehydes also results in depletion of intracellular thiols that sequester the aldehydes, particularly the major intracellular antioxidants glutathione and cysteine (Wood et al. 2007b). Clinically, this reduction in cellular thiols has been reported in schizophrenia (Wood and Wood 2013).

There have been reports of elevated levels of malondialdehyde and acrolein in the plasma, serum, erythrocytes, and brains of AD patients (Casado et al. 2008; Greilberger et al. 2008; Gustaw-Rothenberg et al. 2010; Lovell et al. 2001; Marcus et al. 1998; Martin-Aragon et al. 2009; Nam et al. 2010; Padurariu et al. 2010; Sinem et al. 2010). Levels of 4-HNE have been reported to be increased in brain tissue and cerebrospinal fluid (CSF) of AD patients, and this aldehyde has been found to be present in the neurofibrillary tangles and senile plaques of AD (Zarkovic 2003). Increases in levels of reactive aldehydes including acrolein and 4-HNE have also been reported in the central nervous system of individuals showing early signs of AD (Bradley et al. 2010; Moghe et al. 2015; Williams et al. 2006), suggesting that perhaps an early event in the development of AD could be accumulation of reactive aldehydes. Such aldehydes have been found to play a potential role in prominent aspects of AD. Malondialdehyde, formaldehyde, and methylglyoxal have been reported to increase the rate of amyloid-β (Aβ) oligomer and protofibril formation and to increase the size of the aggregates (Chen et al. 2006). Chronic exposure of rats to acrolein has been reported to result in mild cognitive decline with neuronal loss and activation of astrocytes in the hippocampus; these researchers also observed upregulation of cortical levels of β-secretase (BACE-1, the enzyme catalyzing formation of Aβ from APP) and downregulation of levels of α-secretase [A disintegrin and metalloproteinase domain-containing protein 10; (ADAM-10), responsible for production of a non-amyloidogenic peptide fragments from APP] in the hippocampus and cortex (Huang et al. 2013). Khoramjouy et al. (2020) reported that chronic oral administration of acrolein to rats resulted in impaired learning and memory and that there was a direct correlation between that impairment and the CSF levels of acrolein. Hyperphosphorylation of *tau* protein and acceleration of *tau* aggregation into fibrils have been reported to be induced by acrolein and methylglyoxal (Gomez-Ramos et al. 2003; Kuhla et al. 2007; Li et al. 2012). In late stage AD, when oxidative stress is very advanced, increases in levels of malondialdehyde, acrolein and 4-HNE are very evident (Bradley et al. 2010; Casado et al. 2008).

Elevated malondialdehyde levels have been observed in plasma and cerebrospinal fluid of PD patients (Baillet et al. 2010; Chen et al. 2009; Ilic et al. 1999; Serra et al. 2009), and 4-HNE and malondialdehyde adducts have been found in Lewy bodies in neocortical and brain stem neurons in PD (Zarkovic 2003). Increased malondialdehyde and acrolein content in the substantia nigra has also been reported in PD (Dexter et al. 1989; Shamoto-Nagai et al. 2007). Reactive aldehyde accumulation may be an early step in disease development in PD since increased levels of malondialdehyde adducts in several brain regions of individuals with early stages of PD neuropathology have been reported (Dalfo et al. 2005). Acrolein was found to be colocalized with α-synuclein in substantia nigra neurons of PD patients; the acrolein was shown to enhance α-synuclein oligomerization in vitro in dopaminergic neurons, which can result in mitochondrial dysfunction (Shamoto-Nagai et al. 2007). In addition, Dalfo and Ferrer (2008) discovered malondialdehyde-modified α-synuclein in the substantia nigra and frontal cortex of PD patients, and of individuals with pre-clinical PD.

A key feature of several psychiatric (Buckley 2019; Troubat et al. 2020; Zuliani et al. 2007) and neurodegenerative (Phani et al. 2012; Schain and Kreisl 2017; Wood et al. 1993; Wood 2003) diseases is sustained neuroinflammation that is hypothesized to lead to neuronal dysfunction. It remains to be determined if reactive aldehydes are initiators of these inflammatory cascades or are stimulated by the cascades and thereby contribute to the activated immune response.

**Fig. 4:**
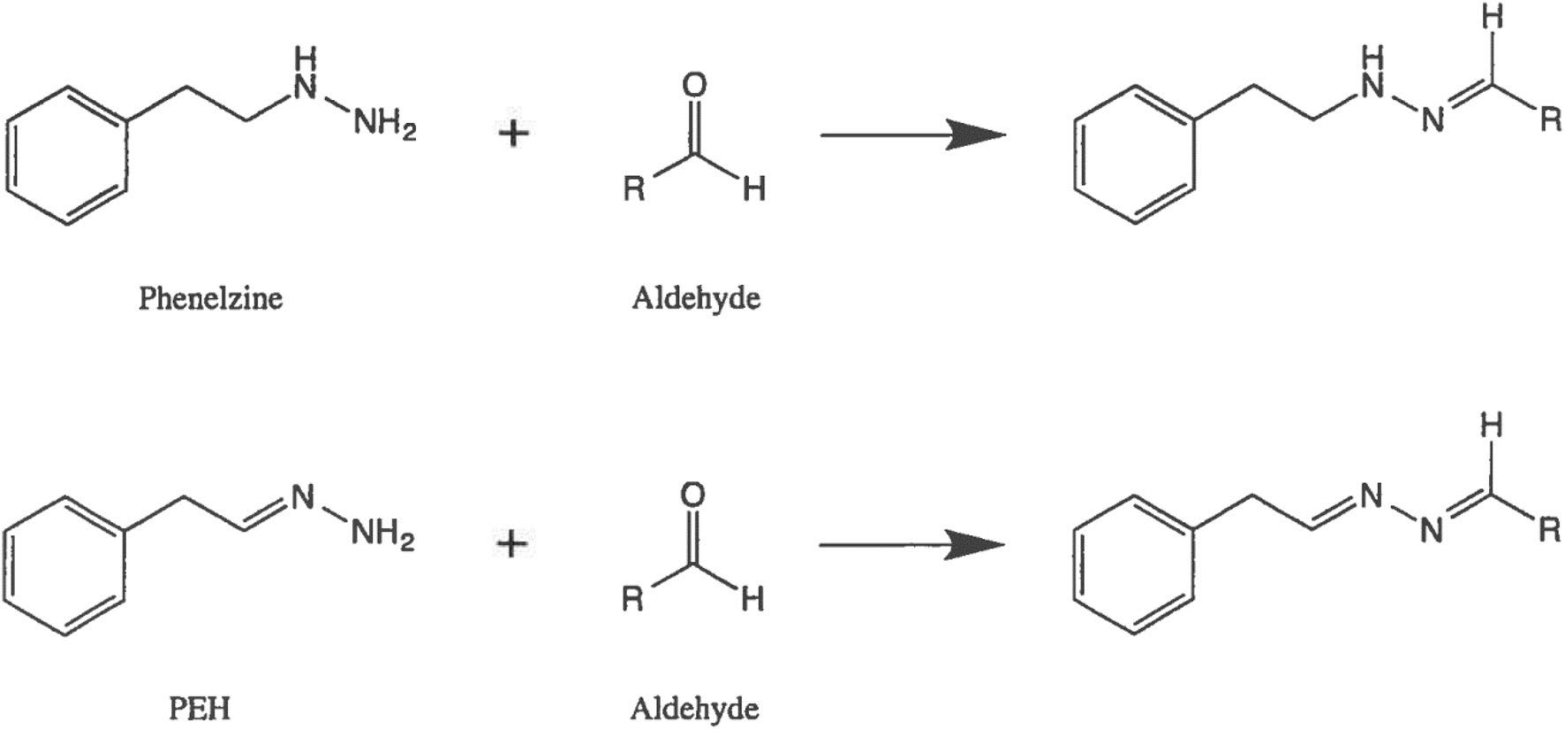
General scheme for reaction of PLZ and PEH with aldehydes

Hydroxylamines and mercapto compounds are known to “mop up” or sequester reactive aldehydes through a chemical reaction (Wood et al. 2006a), but several of these compounds are toxic in their own right. Hydrazines with an unsubstituted NH_2_ group are also known to react with aldehydes (Fig. 4) to produce non-toxic hydrazones. PLZ and PEH are such hydrazines and would be expected to be useful drugs for reducing levels and toxicity of such aldehydes.

**Fig. 5:**
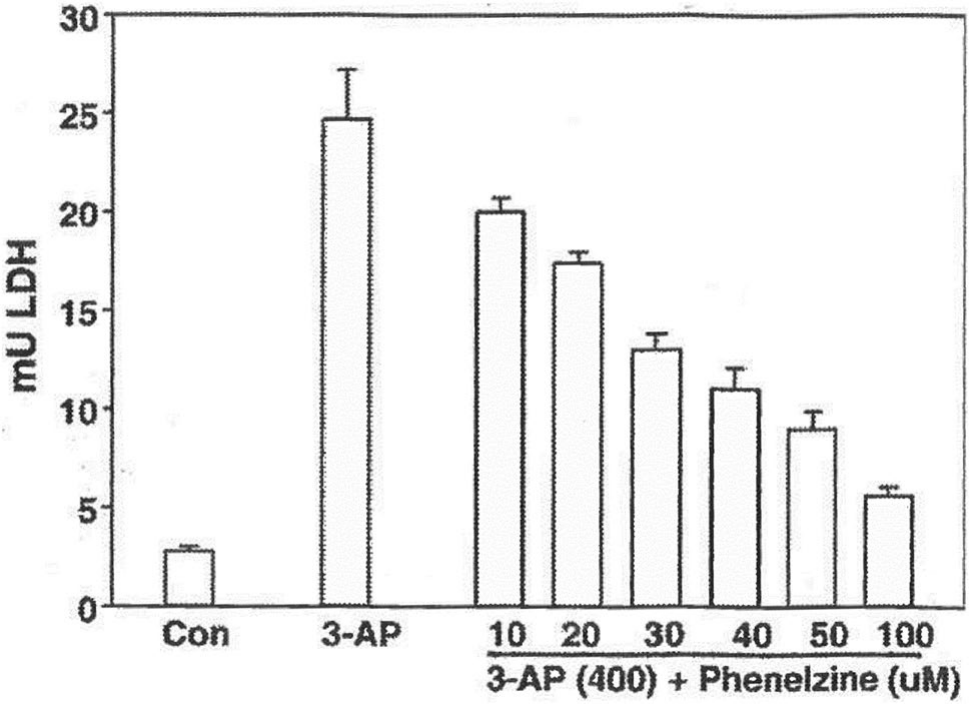
Results from a concentration–response study on effects of PLZ on 3-AP-induced toxicity in rat retinal ganglia cells (from Wood et al. 2006a with permission from Elsevier). PLZ was added as a cotreatment with 3-AP; media were assayed for LDH 24 h later

**Fig. 6:**
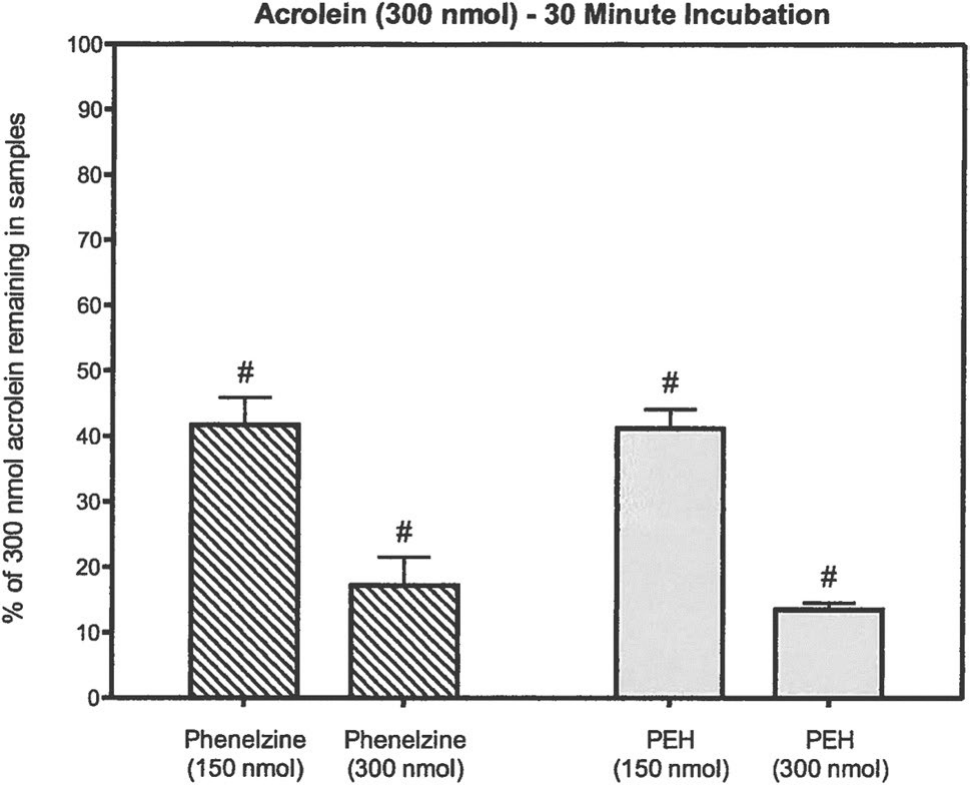
Reduction of acrolein levels in vitro by PLZ and PEH (from Matveychuk 2015, with permission). Acrolein and PLZ or PEH were incubated in phosphate-buffered saline, pH 7.4, for 30 min. Following incubation, 200 µl samples were adjusted to pH 4 and 50 µl of *O*-(2,3,4,5,6-pentafluorobenzyl)-hydroxylamine HCl was added to give a concentration of 20 mM. After 60 min of incubation at room temperature, the samples were acidified and the derivatized aldehyde was extracted with 300 µl hexane. A portion of the hexane layer was used for GC–MS analysis. Data are normalized relative to controls and represent means ± SEM (*N* = 5). All values significantly differ from controls

We conducted a study on the effects of PLZ on the toxic actions of 3-AP and acrolein in retinal ganglion cell cultures and found it to be a potent neuroprotective agent and we have demonstrated, using mass spectrometry, that it does sequester 3-AP under the incubation conditions used (Wood et al. 2007a; Fig. 5). In other studies, we found that PLZ and PEH attenuate the reduction in viability of cultured mouse cortical neurons produced by acrolein (Baker et al. 2019) and sequester several reactive aldehydes in vitro including formaldehyde, acrolein, malondialdehyde, and 4-HNE (MacKenzie 2009; Matveychuk 2015; Fig. 6).

Song et al. (2010) conducted a comprehensive study on the effects of PLZ on formaldehyde-induced toxicity to primary cortical neurons and cortical astrocytes in vitro. Formaldehyde inhibited glutamate uptake by decreasing expression of glutamate transporters in astrocytes and activated the second messenger p38 mitogen-activated protein kinase (p38 MAPK), which participates in a signaling cascade modulating cellular responses to cytokines and stress, and these effects were attenuated by PLZ. In neurons, formaldehyde activated p38MAPK and decreased activation of AKT (a marker of neuronal survival), and PLZ reversed these effects (Song et al. 2010). PLZ has also been reported to attenuate 4-HNE-induced mitochondrial dysfunction in a rat model of traumatic brain injury (Singh et al. 2013) and to mitigate 4-HNE-induced damage to proteins and lipids in blood plasma (Mustafa et al. 2018b).

## Inhibition of Primary Amine Oxidase (PrAO) by PLZ and PEH

**Fig. 7:**
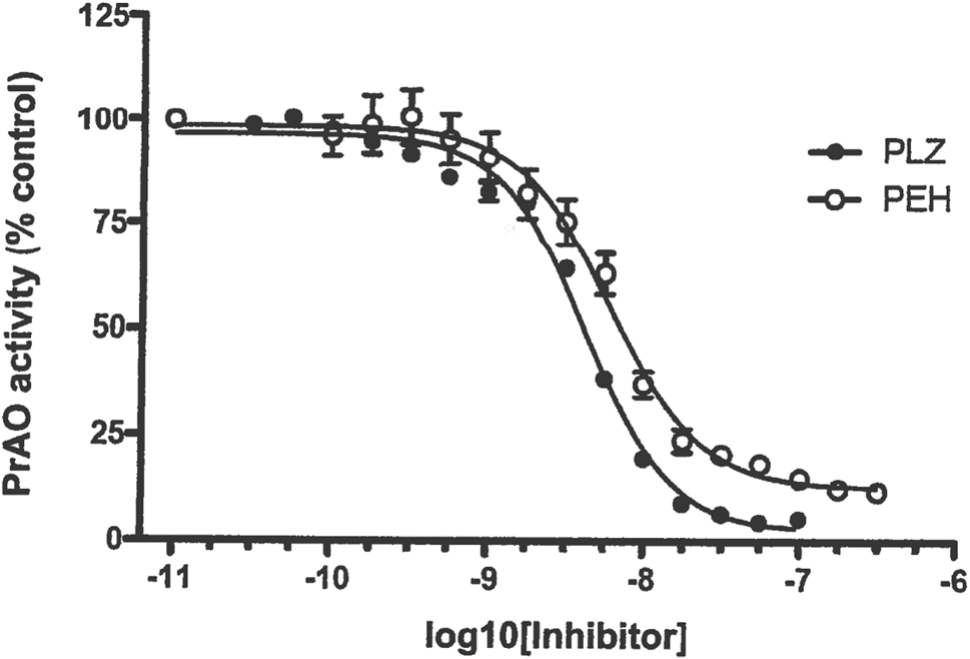
Inhibition by PLZ and PEH of PrAO from a human source (from MacKenzie 2009, with permission). A. Holt (University of Alberta) purified soluble human PrAO from CHO cells over-expressing the human enzyme. PrAO activity was measured using a modification of the spectrophotometric assay of Holt and Palcic (2006), with methylamine as the substrate for the enzyme. Values are expressed as % of control PrAO activity (means ± SEM, *N* = 3)

PrAO is a copper-containing transmembrane glycoprotein, and the extracellular domain may be cleaved off, resulting in a circulating form in plasma (Shanahan et al. 2019; Stolen et al. 2004). In some tissues, the membrane form is synonymous with Vascular Adhesion Protein (VAP-1) involved in migration of leukocytes at sites of inflammation (Pannecoeck et al. 2015; Salmi and Jalkanen 2001; Shanahan et al. 2019). The aldehydes formaldehyde and methylglyoxal are formed by deamination of methylamine and aminoacetone catalyzed, respectively, by the circulating plasma form and the membrane-associated form of PrAO (Lyles 1996; Shanahan et al. 2019). These aldehydes have been shown to increase the formation of β-amyloid (Aβ) β-sheets and fibrillogenesis (Chen et al. 2006), both of which are proposed to be major contributing factors to the pathology of AD (Selkoe 2001; Tanzi and Bertram 2005). Increased serum PrAO activity has been reported in AD patients (Chen et al. 2006; del Mar et al. 2005; Ferrer et al. 2002; Gubisne-Haberle et al. 2004; Pannecoeck et al. 2015; Unzeta et al. 2007; Valente et al. 2012; Yu et al. 2003), and Jiang et al. (2008) reported a strong expression of PrAO colocalized with Aβ deposits on blood vessels of postmortem brain samples from AD patients. Elevated PrAO plasma levels have also been reported in patients with active relapsing multiple sclerosis (Airas et al. 2008), diabetes, and inflammation (Pannecoeck et al. 2015), and inhibition of PrAO has been reported to be of benefit in the relapsing experimental autoimmune encephalomyelitis (EAE) animal model of multiple sclerosis (O’Rourke et al. 2007) and to have anti-inflammatory actions that are beneficial to vascular health (Jarnicki et al. 2016). Ischemia–reperfusion injury in a mouse model of stroke is attenuated in PrAO-deficient mice and by PrAO inhibitors (Kiss et al. 2008). Additionally, inhibition of PrAO has been reported to provide anti-inflammatory protection in a mouse model of intracerebral hemorrhagic stroke (Ma et al. 2011). Horváth et al. (2017) reported that an inhibitor of PrAO had analgesic and anti-inflammatory effects in a mouse model of chronic arthritis. It is of interest that PLZ has also been shown to be a strong inhibitor of PrAO (Lizcano et al. 1996; Lyles 1996); it has been demonstrated in our group that PEH is also a potent inhibitor in vitro of PrAO from human sources (MacKenzie 2009; Fig. 7) and that ip injection of PLZ or PEH increases rat brain levels of methylamine, an indirect indicator of reduction of formaldehyde levels (Matveychuk et al. 2013). These findings and the observations regarding the other actions of PEH mentioned above in this review paper suggest that studies on the effects of PLZ and PEH on animal models of AD are warranted.

## Other Potential Applications of PLZ Based on Neuroprotection

### PLZ and Traumatic Brain Injury and Spinal Cord Injury

Traumatic brain injury (TBI) has been reported to result in mitochondrial dysfunction and induction of lipid peroxidation, the latter resulting in production of reactive aldehydes such as acrolein and 4-HNE, which probably accumulate because of their reduced clearance from damaged neurons (Hill et al. 2017, 2018, 2019). Singh et al. (2013) found that pretreatment with PLZ reduced the inhibitory effects of 4-HNE on mitochondrial complex I and II respiration and also produced a reduction of 4-HNE levels in mitochondria. They also found that PLZ administered to rats 15 min after controlled cortical impact-TBI (CCI-TBI) prevented the decrease in respiratory control ratio produced by the CCI-TBI. Further, they reported that PLZ increased the amount of spared cortical tissue from 86 to 97% and concluded that the neuroprotective actions of PLZ were the result of protection of mitochondria by scavenging 4-HNE. PLZ pretreatment has been reported by Cebak et al. (2017) to prevent both mitochondrial dysfunction and oxidative modification of mitochondrial proteins produced in isolated non-injured rat brain cortical mitochondria by 4-HNE or acrolein. Pargyline, an MAOI which does not have a hydrazine moiety, had no protective effects in a similar study, and the authors concluded that the response to PLZ was related to carbonyl scavenging rather than to inhibition of MAO (Cebak et al. 2017). In a parallel in vivo study, these researchers found that PLZ given 15 min after the CCI-TBI resulted in a reduction of mitochondrial respiratory dysfunction and an increase in cortical tissue sparing.

Kulbe et al. (2018) examined the effects of continuous infusion (for 72 h) of PLZ following severe CCI-TBI in rats and found that it attenuated mitochondrial levels of 4-HNE and acrolein and maintained the mitochondrial respiratory control ratio and cytoskeletal integrity. In a recent study, Hill et al. (2020) reported that administration of PLZ to young adult male rats after severe CCI-TBI resulted in a preservation of both synaptic and non-synaptic mitochondrial bioenergetics observed 24 h later, and that the protection was partially maintained at 72 h.

Acrolein has been proposed to be involved in the propagation of neuropathic pain in spinal cord injury (SCI) by activation of the transient receptor potential ankyrin 1 (TRPA1) cation channel. Lin et al. (2018) reported that PLZ improved post-SCI hypersensitivity and motor neuron survival, decreased acrolein levels and suppressed TRPA1 upregulation in a rat model of ischemia–reperfusion SCI. PLZ has also been reported to reduce the hyperalgesic effects of acrolein inhalation in a rat model of contusion SCI (Butler et al. 2017). Chen et al. (2016b) found reduced neuropathic pain after injury in a rat model of SCI when PLZ was administered by acute, delayed and chronic dosage schedules. It is relevant that hydralazine, another hydrazine drug that, like PLZ, scavenges acrolein, also reduces neuropathic pain and provides neuroprotection in SCI (Due et al. 2014; Liu-Snyder et al. 2006; Park et al. 2015). Chen et al. (2016b) found that PLZ and hydralazine each produced a dose-dependent reduction in levels of acrolein in vivo and that PLZ facilitated recovery of locomotor function and neuroprotection of spinal cord tissue when given for 2 weeks after injury.

### PLZ and Multiple Sclerosis

As mentioned previously in this review, in relapsing multiple sclerosis elevated PrAO activity has been observed in patients (Airas et al. 2008), and improvement of symptoms has been reported in the EAE model after inhibition of PrAO (O’Rourke et al. 2007). Accumulation of toxic aldehydes in plasma, CSF and brain tissue from multiple sclerosis patients has been reported (Bizzozero et al. 2005; Calabrese et al. 1994; Hunter et al. 1985). Studies on the possible role of acrolein in multiple sclerosis have received considerable interest in more recent years (Leung et al. 2011; Tully et al. 2018). Acrolein induces damage to myelin in the spinal cord of mammals (Shi et al. 2011, 2015), and there have been reports of elevated levels of acrolein in the EAE mouse model of multiple sclerosis (Leung et al. 2011; Tully et al. 2018). Hydralazine, which like PLZ is a carbonyl scavenger, has been reported to improve symptoms (Leung et al. 2011) and reduce spinal cord levels of acrolein in the EAE model (Tully et al. 2018). In studies on chronic administration of PLZ in this same model, Musgrave et al. (2011) found that daily injection of PLZ to female mice with EAE prior to onset of clinical signs delayed onset and severity of symptoms and resulted in enhanced exploratory activity, improved depression-like symptoms and a normalization of levels of 5-HT in the ventral horn of the spinal cord. The same research group later found that PLZ administration to the EAE mice even after the onset of clinical signs can reduce the severity of these signs and improve exploratory activity (Benson et al. 2013). Potter et al. (2016) found an antinociceptive effect and normalization of primary somatosensory cortex neural ensemble responses, neuronal morphology and cortical microglia numbers as well as attenuation of reactivity of the VGLUT1 glutamate transporter in EAE mice treated with PLZ. Antinociceptive effects have also been studied in models other than EAE. Mifflin et al. (2016) studied the effects of PLZ and PEH in the formalin model of tonic nociception and found that both PEH and PLZ reduced nociceptive behaviors in the second phase of pain induction; interestingly, the reduction was similar in both sexes of mice in the case of PEH, but much more prominent in male mice in the case of PLZ. Potter et al. (2018) found that PLZ selectively inhibits ongoing inflammatory pain but spares transient reflexive and acute nociception; these same researchers reported that PLZ reduced intracellular calcium responses to superfusion of glutamate ex vivo in lumbar spinal cord slices. As mentioned earlier in this review paper, PLZ has been reported to reduce pain in TBI and SCI, and sequestration of acrolein is thought to play a role in that action of PLZ. Similar effects of PEH and PLZ on acrolein in multiple sclerosis are certainly feasible, but, to our knowledge, this possibility has not been studied to date. Similarly, we are not aware of any direct comparisons of long-term administration of PLZ and PEH in animal models of multiple sclerosis. Such studies are warranted in this and other animal models of neurodegenerative disorders to determine whether the GABA levels gradually decrease over time in response to PLZ since less PEH may be formed as PLZ-induced inhibition of MAO increases, oxidation of PLZ to PEH decreases, and inhibition of GABA-T is consequently reduced.

### PEH as an Anticonvulsant?

To our knowledge, PEH has not been tested as an anticonvulsant in vivo in animal models, but the literature on PEH suggests that such studies are warranted. In a physiological study, Duffy et al. (2004) applied solutions of PEH to rat hippocampal slices and found a 60% increase in GABA levels in the slices. Further, they observed that hyper-excitation during epileptiform bursting induced by superfusion with Mg^2+^-free or high-K^+^ artificial CSF was reduced, results suggesting a potential anticonvulsant action of PEH. The GABA-T-inhibiting/GABA-elevating action of PEH in rat brain ex vivo also suggests that PEH should be investigated as a potential anticonvulsant. Vigabatrin is a GABA-T inhibitor marketed as an anticonvulsant, but studies in rats ex vivo indicated that both PLZ and PEH are much more potent than vigabatrin at inhibiting GABA-T and elevating brain GABA levels in rats (MacKenzie et al. 2008a; Todd and Baker 2008). In addition, PLZ and PEH can sequester reactive aldehydes such as malondialdehyde, acrolein and 4-HNE that have been implicated in the etiology of some types of epilepsy (Cardenas-Rodriguez et al. 2013; Hogard et al. 2017; Lorigados Pedre et al. 2018; Olowe et al. 2020).

## Other Neurobiological Findings with PLZ that may Have Relevance to Neuroprotection

The reactive nitrogen species (RNS) peroxynitrite is produced by the fusion of the superoxide anion and nitric oxide. Peroxynitrite is thought to produce its adverse effects after decomposing to yield a nitrogen dioxide radical, a hydroxyl radical and a nitryl cation, all of which can cause damage to nerve cells (Bedard and Krause 2007; Mustafa et al. 2018a). Peroxynitrite has been linked to the etiology of disorders such as diabetes, hypertension and atherosclerosis and may contribute to aging (Finkel and Holbrook 2000; Niemann et al. 2017; Sugamura and Keaney 2011). Mustafa et al. (2018a) reported that PLZ protected against protein carbonyl formation, protein nitration and lipid peroxidation in peroxynitrite-treated plasma and platelet samples. The authors concluded that the ability of PLZ to scavenge reactive aldehydes was responsible for its protective effects in all three of these oxidative stress situations (Mustafa et al. 2018a).

The dopamine system neurotoxin 1-methyl-4-phenyl-1,2,3,6-tetrahydropyridine (MPTP) is converted metabolically to its metabolite 1-methyl-4-phenylpyridinium (MPP^+^) by MAO-B, and this metabolite has been proposed to cause cell death by opening the mitochondrial permeability transition pore (Cassarino et al. 1999; Lee et al. 2003). Lee et al. (2003) studied the effects of MPP^+^ on differentiated PC12 cells and reported that PLZ reduced MMP^+^-induced condensation and fragmentation of nuclei and also counteracted the decrease in mitochondrial membrane potential, as well as cytochrome c release, formation of reactive oxygen species, depletion of total glutathione levels, and cell death induced by H_2_O_2._

Actions of PLZ on brain-derived neurotrophic factor (BDNF) may also contribute to the neuroprotective properties of PLZ. Chronic, but not acute, administration of a number of antidepressants, including PLZ, has been reported to produce an increase in rat brain levels of BDNF (Balu et al. 2008; Nibuya et al. 1995). In a study in rats, Dwivedi et al. (2006) reported that chronic (21 days) administration of PLZ resulted in increased mRNA expression of BDNF in frontal cortex and hippocampus and reversed the corticosterone-induced decrease in the expression of BDNF in the same brain areas. In a study on mouse hippocampus, Fred et al. (2019) investigated the effects of ip injection of the antidepressants PLZ, fluoxetine, imipramine and ketamine on the coupling of the tropomyosin-related kinase B (TRKB) receptor and the adaptor protein complex-2 (AP-2) involved in vesicular endocytosis and suggested a novel mechanism for all of these antidepressants in which they disrupt the TRKB:AP2M subunit interaction and thereby promote TRKB cell surface exposure and BDNF signaling.

The neural cell adhesion molecule L1CAM (L1) plays a functional role in the developing and adult nervous system and is thought to be linked to several neurodegenerative diseases (Joseph et al. 2020). In the injured spinal cord, L1 can promote axonal regrowth and enhance survival of neurons, synaptic plasticity and remyelination (Li et al. 2018). In studies in a zebrafish SCI model, Li et al. (2018) found that addition of PLZ to the aquarium water resulted in an accelerated recovery of the reduced locomotor activity produced by the spinal cord transection and promoted axonal regrowth and remyelination in both larval and adult zebrafish. In the same study, these researchers proposed that PLZ was a L1 mimetic since it upregulated expression and proteolysis of L1 and phosphorylation of extracellular-signal-regulated-kinase (Erk) caudal to the site of the lesion in the spinal cord. In another study in zebrafish, Joseph et al. (2020) reported that PLZ counteracted the toxicity induced by the environmental neurotoxin paraquat—they found that PLZ prevented the reduction in tyrosine hydroxylase activity and dopamine levels, reduced generation of reactive oxygen species, protected against impairment of mitochondrial viability, enhanced the antioxidant system and prevented a decrease in levels of adenosine triphosphate (ATP).

Phosphorylation of Fas-associated death domain (FADD), an adaptor of death receptors, can result in induction of anti-apoptotic actions (García-Fuster and García-Sevilla 2016). It has been reported that acute administration of PLZ to rats resulted in a marked increase in the ratio of phosphorylated to non-phosphorylated FADD in brain cortex, and the authors suggested that this potential anti-apoptotic action of PLZ may be related to its GABA-elevating action and subsequent activation of GABA_A_ receptors. However, the effect on the p-FADD/FADD ratio was not evident after chronic administration (14 days) of PLZ, perhaps because MAO-mediated generation of PEH from PLZ cannot be maintained under conditions of substantial MAO inhibition.

In metabolomics studies, we also found that ip treatment of male rats with PLZ or PEH resulted in marked increases in brain cortex levels of ornithine (MacKenzie et al. 2008b) and a number of N-acetylated amino acids (Wood et al. 2020). We speculated that the increased ornithine levels may be an indicator of decreased formation of glutamate and/or polyamines (resulting in decreased formation of reactive aldehydes such as acrolein and 3-AP), thus contributing to neuroprotection. The possible involvement of the effects on *N*-acetylamino acids in neuroprotection awaits a further knowledge of the role of these *N*-acetylamino acids in the central nervous system, although *N*-acetylasparate is present in millimolar concentrations in brain and is a marker for viable neurons (Demougeot et al. 2001), *N*-acetylglutamate is a modulator of the urea cycle, *N*-acetyl-leucine is a modulator of vestibulocerebellar and posterolateral thalamic circuits related to vestibular function (Günther et al. 2015) and *N*-acetylglutamine has been proposed to be involved in the sleep–wake cycle (Bourdon et al. 2018).

## Conclusion

Although marketed initially as an antidepressant because of its ability to inhibit MAO, PLZ is a multifaceted drug with a multitude of actions that may be relevant to neuroprotection and the pharmacotherapy of several psychiatric and neurological disorders. Its anxiolytic effects have been demonstrated clinically, and results from animal models suggest that PLZ and/or its metabolite PEH could be considered for studies as adjunctive agents in disorders such as stroke, AD, PD, SCI, TBI, epilepsy, and multiple sclerosis. PLZ and PEH share abilities to inhibit GABA-T and PrAO and sequester reactive aldehydes. They differ from each other in their ability to inhibit MAO, with PLZ being a very strong irreversible of MAO-A and -B, while PEH is only a weak inhibitor of both of these isoforms of MAO.

This difference in inhibition of MAO suggests that PEH is unlikely to have antidepressant efficacy, but that it is worth investigating in some of the other disorders mentioned above because of its other neuroprotective properties and the fact that the dietary caution recommended with non-selective, irreversible MAOIs would not be required.

## Data Availability

This a review article, but statements are based on the authors’ research. Data can be obtained from the authors on reasonable request.

## Abbreviations

3-AP: 3-Aminopropanal
AD: Alzheimer’s disease
ADAM-10: A disintegrin and metalloproteinase domain-containing protein 10; α-secretase
AP-2: Adaptor protein complex-2
APP/PS1: Amyloid precursor protein/presenilin 1
ATP: Adenosine triphosphate
BACE: β-Secretase
CCI-TBI: Controlled cortical impact traumatic brain injury
CSF: Cerebrospinal fluid
DOPAL: 3,4-Dihydroxphenylacetaldehyde
DOPEGAL: 3,4-Dihydroxyphenylglycolaldehyde
EAE: Experimental autoimmune encephalomyelitis
FADD: Fas-associated drug domain
GABA: γ-Aminobutyric acid
GABA-T: GABA transaminase
H_2_O_2_: Hydrogen peroxide
HIAL: 5-Hydroxyindoleacetaldehyde
HNE: 4-Hydroxy-2-nonenal
L1: Neuronal cell adhesion molecule
MAO: Monoamine oxidase
MPP^+^: 1-Methyl-4-phenylpyridinium
MPTP: 1-Methyl-4-phenyl-1,2,3,6-tetrahydropyridine
PD: Parkinson’s disease
PEH: β-Phenylethylidenehydrazine
PLZ: Phenelzine
PrAO: Primary amine oxidase
ROS: Reactive oxygen species
SCI: Spinal cord injury
SSAO: Semicarbazide-sensitive amine oxidase
TBI: Traumatic brain injury
TRKB: Tropomyosin-related kinase B
TRPA1: Transient receptor potential ankyrin 1
VAP-1: Vascular adhesion protein
VGLUT: Vesicular glutamate transporter
